# Tripartite microbial augmentation of *Bradyrhizobium diazoefficiens*, *Bacillus* sp. MN54, and *Piriformospora indica* on growth, yield, and nutrient profiling of soybean (*Glycine max* L.)

**DOI:** 10.3389/fmicb.2024.1437489

**Published:** 2025-03-07

**Authors:** Munazza Rafique, Muhammad Naveed, Muhammad Zahid Mumtaz, Abid Niaz, Saud Alamri, Sajid ur Rehman, Manzer H. Siddiqui, Adnan Mustafa

**Affiliations:** ^1^Soil Bacteriology Section, Agricultural Biotechnology Research Institute, AARI, Faisalabad, Pakistan; ^2^Institute of Soil and Environmental Sciences, University of Agriculture, Faisalabad, Pakistan; ^3^College of Agronomy, Gansu Agricultural University, Lanzhou, China; ^4^Institute of Molecular Biology and Biotechnology, The University of Lahore, Lahore, Pakistan; ^5^Department of Botany and Microbiology, College of Science, King Saud University, Riyadh, Saudi Arabia; ^6^Agricultural Biotechnology Research Institute, AARI, Faisalabad, Pakistan; ^7^Key Laboratory of Vegetation Restoration and Management of Degraded Ecosystems, South China Botanical Garden, Chinese Academy of Sciences, Guangzhou, China

**Keywords:** triple inoculation, nodulation, *Bradyrhizobium diazoefficiens*, *Piriformospora indica*, *Bacillus* spp., pulses

## Abstract

**Introduction:**

Enhancing productivity and nutrient content of soybean (*Glycine max* L.) is vital for sustainable agriculture. The utilization of beneficial bacterial and fungal strains has shown promising results in promoting plant growth and improving nutrient uptake. However, the effects of the individual and interactions of such microbes on soybean growth, yield, and nutrient profiling remain inadequately understood. Thus, there is a pressing need to comprehensively investigate the impact of tripartite microbial augmentation on soybean cultivation.

**Methods:**

This field study aims to elucidate the synergistic mechanisms underlying the interactions between *Bacillus* sp. MN54, *Bradyrhizobium diazoefficiens*, and *Piriformospora indica* and their collective influence on soybean growth parameters, yield and nutritional quality.

**Results:**

*In vivo* compatibility tests revealed that consortium applications led to a maximum of 90% soybean germination. The field study demonstrated a significant increase in plant height (17.01%), nodules plant^–1^ (17.35%), pods plant^–1^ (12.11%), and grain yield (20.50%) due to triple inoculation over untreated control. The triple inoculation also significantly increased chlorophyll a, b, and leghemoglobin contents by 19.38, 21.01, and 14.28%, respectively, compared to control. Triple inoculation promoted crude fiber, protein, and oil content by 14.92, 8.78, and 10.52%, respectively, compared to the untreated control. The increase in nitrogen content by 7.33% in grains and 6.26% in stover and phosphorus by 11.31% in grains and 12.72% in stover was observed through triple application over untreated control.

**Discussion:**

Our findings highlight the potential of microbial inoculants as biofertilizers in sustainable soybean production. The triple inoculation with *Bacillus* sp. MN54, *Bradyrhizobium diazoefficiens*, and *Piriformospora indica* significantly improved soybean growth, yield, grain quality attributes, and nutrient uptake. This microbial consortium application could help to enhance agricultural productivity by boosting the nodulation in soybean and improving synergism between the microbial strains.

## 1 Introduction

Rapid population growth and climate change pose significant challenges to global food production, and cause natural resource scarcity. As the world’s population approaches 9 billion by 2050, addressing malnutrition becomes increasingly urgent (Kumar and Dubey, 2020). To maximize the produce from current agricultural land, agricultural pressure generated by population increase has led to extensive usage of chemical fertilizers and pesticides, and among them, about 20–30% of the fertilizers are absorbed by the plants (Saeed et al., 2021). Inefficient nutrient usage in agriculture and soil dynamics results in more than half of fertilizers being lost to the environment (Fageria, 2014). Unfortunately, nitrogen (N) fertilizers given to the soil are easily volatilized, washed away, while Phosphorus (P) fertilizers gets fixed and gradually changed into unavailable forms by natural processes, putting the ecology and biodiversity at greater risk. As a result, total agricultural productivity has decreased, and environmental issues such as habitat loss, carbon emissions, water contamination, and soil pollution have occurred (Zou et al., 2022). Farmers interests are likewise impacted by increasing agricultural input costs and lowering their profits. This situation demands alternative approaches to enhance the efficiency of applied fertilizers and ensure environmental sustainability.

Microbes, particularly those associated with plant roots, collectively termed the plant microbiome, emerge as pivotal allies in reducing the chemical fertilizer demand (Bender et al., 2016). Among these, beneficial microbes known as Plant Growth-Promoting Rhizobacteria (PGPR) and mycorrhizal fungi have been extensively documented for their ability to enhance plant nutrient uptake, promote growth through hormonal interactions, and increase resistance to abiotic stresses such as drought and salinity (Khan et al., 2016; Rajkumar et al., 2017). The mechanisms by which these microbes operate not only underline the potential to overcome nutrient deficiency but also aid crops in adapting to adverse environmental conditions. Furthermore, combining microbial solutions offers a dual benefit: sustaining crop yields and mitigating adverse environmental impacts.

Among leguminous crops, soybean (*Glycine max*) is one of the most crucial crops globally, valued for its high protein content and role in sustainable agriculture through nitrogen fixation. The symbiotic relationship between soybean plants and the nitrogen-fixing bacteria is well-documented, facilitating significant reductions in nitrogen fertilizer requirements (Hungria and Mendes, 2015). Still, the native nitrogen-fixing bacteria usually have nutritional competitiveness and poor survival rates, making them unable to ensure adequate nodulation and sufficient biological nitrogen fixation (Masson-Boivin and Sachs, 2018). One of the methods for increasing biological nitrogen fixation is inoculating seed with biological nitrogen-fixing bacteria. However, further enhancement of soybean productivity may be achieved through co-inoculation with other beneficial soil bacteria, such as *Bacillus* species (Solanki et al., 2023). This integrative approach leverages the combined effects of nitrogen-fixing bacteria, known for its nitrogen-fixing capabilities, and *Bacillus*, recognized for promoting plant growth by various mechanisms, including phytohormone production, phosphate solubilization, and antagonistic activity against plant pathogens (Ahmad et al., 2019; Pankievicz et al., 2021). For example, *Bacillus* strains, which are prolific producers of growth-promoting substances and biocontrol agents, can significantly affect root architecture and thereby improve the efficacy of nitrogen-fixing bacteria in the rhizosphere through a healthier root environment (Xie et al., 2016). This co-inoculation strategy not only boosts the overall health and growth rate of soybean but also contributes to a more sustainable and environmentally friendly farming practice to overcome nutrient deficiency (Compant et al., 2010).

Similarly, it has been found that endophytic growth-promoting microorganisms and nitrogen-fixing bacteria species act synergistically to boost the nitrogen-fixation efficiency of lentils (Saini and Khana, 2012). For the past few decades, *Piriformospora indica* (*P. Indica*) has been a famous and most studied endophytic fungus for vegetative increase and plant resistance to nutrient deficiency (Iqbal et al., 2023). *P. Indica* improves host plants’ growth, biomass, and seed yield and confers resistance to various abiotic and biotic stresses (Franken, 2012). The dual and consortium microorganisms have enhanced plant productivity (Varma et al., 2012). It has been found that the effect of single and dual inoculation in legumes was observed in previous studies (Ferreira et al., 2019; Jaiswal and Dakora, 2020; Gupta et al., 2022), however, no study was conducted on multiple inoculation to enhance soybean growth and yield. Therefore, this study aimed to evaluate the synergistic effects of tripartite microbial augmentation of *Bradyrhizobium diazoefficiens* (*B*. *diazoefficiens*), *Bacillus* sp. MN54 and *P. indica* on soybean growth, yield, and nutrient uptake under field situations. This microbial augmentation was evaluated for synergistic effects to improve nodulation in soybean and nutrient uptake.

## 2 Materials and methods

### 2.1 Acquiring endosymbionts and PGPR culture

The microbial cultures of well-characterized plant growth-promoting *Bacillus* sp. MN54 (accession number KT375574) (Naveed et al., 2014; Afzal et al., 2020) was obtained from the Institute of Soil and Environmental Science, University of Agriculture, Faisalabad, Pakistan. *B. diazoefficiens* cultures were obtained from the Japan Collection of Microorganisms,^1^ while *P. Indica* culture was obtained from the German Collection of Microorganisms and Cell Cultures, GmbH.^2^ The subculture of *B. diazoefficiens* was prepared in Yeast Mannitol extract broth (YMEB) at 28 ± 1°C and 120 rpm for five days. The nutrient broth was used to grow *Bacillus* sp. MN54 at 28 ± 1°C and 120 rpm for 48 h. The *P. indica* was grown in potato dextrose broth (PDB) at 28 ± 1°C and 120 rpm for 1 week. The subcultures were kept at 4°C for further use.

### 2.2 Evaluation of the compatibility of endophytes and PGPR under axenic conditions

The compatibility of microbial cultures of *B. diazoefficiens*, *P. indica*, and *Bacillus* sp. MN54 was tested through the streak plate method (Raja et al., 2006) and a germination assay. In the streak plate method, *P. indica* biomass was cut with a sterile cutter, placed in the middle of a plate containing nutrient agar medium, and kept for control. *Bacillus* sp. MN54 and *B. diazoefficiens* were streaked on one side, and the *P. indica* culture block was placed on the opposite side with three replications and kept at 28 ± 2°C in an incubator for five days. After incubation, these cultures were observed for compatibility with each other. During the germination assay, healthy soybean seeds were surface sterilized using 3.0% sodium hypochlorite solution for 2 min and then washed thoroughly with sterilized water. The surface sterilized seeds were treated with (1) *B. diazoefficiens* inoculation, (2) *B. diazoefficiens* + *Bacillus* sp. MN54 inoculation, (3) *B. diazoefficiens + P. indica* inoculation, (4) *B. diazoefficiens, Bacillus* sp. MN54 and *P. indica* inoculation. The 1 mL log phase culture was used with a ratio of 1:1:1 in triple-inoculation. Treated seeds were kept in Petri dishes comprising 0.7% water agar at 28°C in the growth room for seven days, and each treatment was repeated thrice (Wasike et al., 2009).

### 2.3 Effect of endosymbionts and PGPR on soybean cultivation under field conditions

A field experiment was conducted in two consecutive summer seasons of 2021 and 2022 at oilseed Research Institute, AARI, Faisalabad, Pakistan. The soil used in this experiment was sandy clay loam having EC 1.4 dS m^–1^, organic matter 0.67%, pH 7.75, total nitrogen 0.032%, available phosphorus 7.40 mg kg^–1^, and extractable potassium 116 mg kg^–1^. The experiment was designed in a randomized complete block design (RCBD) with 24 plots. The soybean variety “Faisal” was used at the rate of 120 kg ha^–1^ with a row-to-row distance of 30 cm in a plot size of 4.0 m × 1.2 m (4.8 m^2^). The pooled mean of maximum and minimum temperature during the crop growth period was 39.9 and 27.8°C, respectively. The research location has a subtropical climate, receives 25 mm of rainfall annually, and is between latitude 31.4187°N, longitude 73.0791°E, and 186.0 m above sea level. The treatments in terms of seed coating were (1) Control, (2) *B. diazoefficiens*, (3) *Bacillus* sp. MN54, (4) *P. indica*, (5) *B. diazoefficiens* + *Bacillus* sp. MN54, (6) *P. indica* + *Bacillus* sp. MN54, (7) *B. diazoefficiens* + *P. indica*, and (8) *B. diazoefficiens* + *Bacillus* sp. MN54 + *P. indica* in triplicate.

Soybean seeds were inoculated with respective cultures of *B. diazoefficiens*, *Bacillus* sp. MN54 and *P. indica* alone and in different combinations according to the treatment plan. For this purpose, surface sterilized seeds were inoculated with inoculum-based slurry in 1:1 ratio (50% seed and 50% slurry). This slurry was prepared using 50% inoculum, 30% sterilized peat, 10% clay, and 10% sugar solution. At the time of inoculation, the CFU of *B. diazoefficiens* and *Bacillus* sp. was MN54 1 × 10^8^ CFU mL^–1^, while for *P. indica*, CFU was 1 × 10^6^ CFU mL^–1^. In co-inoculation and triple-inoculation, the *B. diazoefficiens*, *Bacillus* sp. MN54 and *P. indica* were applied to soybean seeds at a 1:1 ratio and 1:1:1 ratio, respectively. The number of seeds was uniform in all the experimental plots. Seeds were thoroughly mixed until a thin, fine layer of inoculum appeared. The seeds were spread in the shade to dry overnight in the laboratory. Field was prepared by giving three ploughings followed by planking. The treated seeds were sown in mid-June with a seed rate of 70 kg ha^–1^. After germination, the plant-to-plant distance of 5–6 cm was maintained while the row-to-row distance was 45 cm. All the agronomic practices were adopted for cultivating the soybean crop. The recommended doses of chemical fertilizers used in the experiment were 25–25–50 NPK kg ha^–1^ in the form of Urea, single superphosphate (SSP), and sulphate of potash (SOP), respectively. Half of N, all P, and K doses were mixed with soil at the time of sowing, and the remaining N was applied with the first and second irrigations with equal splits. Observations regarding emergence percentage were recorded after 10 days of sowing. Nodular data was recorded at the flowering stage. Plant height, grain yield, number of pods, and dry biomass were recorded at harvesting. Chlorophyll and leghemoglobin contents were recorded after 90 days of sowing (DAS). After harvesting, nutrient contents, seed protein, fiber, moisture, and oil contents were recorded.

### 2.4 Plant agronomic performance

Ten plants were randomly selected from each replication of a 2-year trial to determine the plant height with the help of a meter rod on a cm scale. Data on emergence count was determined by recording the number of emerged seedlings per meter row length from a central row of each plot after leaving two border rows on each side. Ten randomly selected plants were uprooted from each plot and were sun-dried and then oven-dried at 60°C for two days to record the dry weight of the shoot in grams (g).

Symbiotic dependency (SD) was recorded at 90 DAS. To determine the SD of soybean, the formula given by Gerdemann (1975) was used:


S⁢y⁢m⁢b⁢i⁢o⁢t⁢i⁢c⁢d⁢e⁢p⁢e⁢n⁢d⁢e⁢n⁢c⁢y=

$$\frac{D\,r\,y\,w\,e\,i\,g\,h\,t\,o\,f\,i\,n\,o\,c\,u\,l\,a\,t\,e\,d\,p\,l\,a\,n\,t\,s}{D\,r\,y\,w\,e\,i\,g\,h\,t\,o\,f\,u\,n\,i\,n\,o\,c\,u\,l\,a\,t\,e\,d\,p\,l\,a\,n\,t\,s}\times 100$$

Nodulation parameters, including the number of nodules and the dry weight of nodules, were recorded by taking an average number of nodules carefully detached from ten randomly uprooted plants. The detached nodules were oven-dried at 60°C for two days, and the dry weight of nodules per plant was recorded in g. The grain yield from each plot (Kg m^–2^) was recorded, and the final data was presented in kg ha^–1^.

### 2.5 Physiological attributes

The chlorophyll content of leaves was estimated using the method of Arnon (1949) at 645 and 663 nm wavelengths through a spectrophotometer (Agilent Technologies, Santa Clara, CA, United States). At noon (between 10:00 and 14:00), after 90 DAS, physiological parameters were recorded from entirely green leaves. For chlorophyll a and b content, plant leaves were crushed in acetone and centrifuged at 10,000 rpm for 10 min. Then, the supernatant was subjected to record absorbance of chlorophyll “a” at 645 nm and that of “b” at 663 nm contents through a spectrophotometer (Agilent Technologies, Santa Clara, CA, United States; Arnon, 1949). Root nodules were taken at the flowering stage to determine the volume of pink bacteroid tissue of nodules and a minor section of nodules (5 μL) was made using a sharp cutter. The amount of pink bacteroid tissue comprising the leghemoglobin in the nodule cortex was determined following the method described by Sadasivam and Manickam (2008). According to this method, 1 g of fresh root nodules was taken and crushed in 5 mL of cold phosphate buffer (0.1 M) with the help of a pestle and mortar in an ice-cold environment. The homogenized mixture was centrifuged at 10,000 rpm for 10 min at 4°C, and a supernatant containing leghemoglobin was collected. Further impurities were removed by adding chilled acetone in 4:1 ratio of nodule extract and incubated for 15 min. The mixture was again centrifuged at 10,000 rpm for 10 min at 4°C. The supernatant was discarded, and the precipitate was dissolved in a phosphate buffer. The absorbance of this mixture was read at 540 nm using a spectrophotometer (Agilent Technologies, Santa Clara, CA, United States), and leghemoglobin concentration was calculated using the following formula. The molar extinction coefficient of leghemoglobin was taken around 11 mM cm^–1^, and the path length (1 cm) refers to the distance that light travels through the sample in the spectrophotometer cuvette.


$$L\,e\,g\,h\,e\,m\,o\,g\,l\,o\,b\,i\,n\,\left(m\,g\,g^{-1}\right)=$$

A⁢b⁢s⁢o⁢r⁢b⁢a⁢n⁢c⁢e⁢a⁢t⁢ 540⁢n⁢mM⁢o⁢l⁢a⁢r⁢e⁢x⁢t⁢i⁢n⁢c⁢t⁢i⁢o⁢n⁢c⁢o⁢e⁢f⁢f⁢i⁢e⁢n⁢t×P⁢a⁢t⁢h⁢l⁢e⁢n⁢g⁢t⁢h×S⁢a⁢m⁢p⁢l⁢e⁢w⁢e⁢i⁢g⁢h⁢t

### 2.7 Assessment of seed quality and nutrient acquisition

Seeds were carefully cleaned and ground to pass through a 0.4 mm screen. AOAC. (1975) method was followed for proximate analysis to determine crude fiber, protein, and moisture content. The protein contents were determined by converting total nitrogen content using a specific factor of 6.25, using the Kjeldahl method. The moisture content in seeds was determined by oven drying 5 g of seeds at 105°C until they reached a constant weight, which was calculated using the following formula.


$$Moisturecontent\left(\%\right)=\frac{I\,n\,i\,t\,i\,a\,l\,w\,e\,i\,g\,h\,t-F\,i\,n\,a\,l\,w\,e\,i\,g\,h\,t}{I\,n\,i\,t\,i\,a\,l\,w\,e\,i\,g\,h\,t}\times 100$$

For crude fiber determination, 2 g of dried ground seeds were acid-digested using 1.25% sulfuric acid. The mixture was boiled for 30 min, filtered, and washed with hot distilled water to neutralize the pH. Further alkali digestion was performed through 1.25% sodium hydroxide and boiled for 30 min. The mixture was again washed with distilled water to neutralize the pH. The residue samples were oven-dried at 105°C until constant weight. The crude fiber of residue samples was performed through dry ashing in a muffle furnace at 550°C for 2 h to remove organic matter, and samples were weighed to calculate crude fiber using the following formula.


Crudefiber(%)=

$$\frac{W\,e\,i\,g\,h\,t\,o\,f\,d\,r\,i\,e\,d\,r\,e\,s\,i\,d\,e-W\,e\,i\,g\,h\,t\,o\,f\,d\,r\,y\,a\,s\,h}{W\,e\,i\,g\,h\,t\,o\,f\,s\,a\,m\,p\,l\,e}\times 100$$

Seed oil content was determined using Soxhlet’s apparatus as described in AOAC. (1984). This method extracted 5 g oven-dried seed samples using hexane solvent in the Soxhlet apparatus. A rotary evaporator was used to recover oil from the solvent-oil mixture. The extracted oil was dried at 105°C for 30 min, and oil contents were calculated using the following formula.


$$Oilcontents\left(\%\right)=\frac{W\,e\,i\,g\,h\,t\,o\,f\,e\,x\,t\,r\,a\,c\,t\,e\,d\,o\,i\,l}{W\,e\,i\,g\,h\,t\,o\,f\,s\,a\,m\,p\,l\,e}\times 100$$

The total nitrogen (N) content of shoot and grain was determined using Kjeldahl’s McKenzie and Wallace (1954) technique with slight modification. Estimation of total phosphorous (P) content of shoot in straw and seed was carried out by digestion using a triacid mixture (HNO_3_: HClO_4_: H_2_SO_4_) (v/v) (Jackson, 1973). Micronutrient contents like iron (Fe), Zinc (Zn), and manganese were determined by following the method of Estefan et al. (2013).

### 2.8 Statistical analysis

The collected data in triplicate was subjected to analysis of variance (ANOVA) in Randomized Complete Block Design (RCBD) through the software Statistix version 8.1. The Least Significant Difference (LSD) test (Steel et al., 1997) was employed to compare the mean values of each attribute at *p* ≤ 0.05. The correlation matrix and principal component analysis were performed using R software version 4.1.2.

## 3 Results

### 3.1 *In vitro* and *in vivo* experiments demonstrated the compatibility of triple inoculation

From the streak plate method, it was observed that *B. diazoefficiens and Bacillus* sp. MN54 showed a synergistic effect against *P. indica*. Moreover, during the germination assay, germination was increased by triple inoculations (*B. diazoefficiens*, *Bacillus* sp. MN54, and *P*. *indica*) than by inoculation *B. diazoefficiens* alone. A maximum increase in germination (90%) was observed by triple inoculations over inoculation with *B. diazoefficiens* alone (Table 1).

**TABLE 1:** Compatibility test of *Bradyrhizobium diazoefficiens*, *Bacillus* sp. MN54 and *Piriformospora indica* on seed germination assay under axenic conditions.

Treatments	Germination (%)
Control	60 ± 2.24 e
*B. diazoefficiens*	70 ± 2.95 d
*B. diazoefficiens + Bacillus* sp. MN54	81 ± 3.64 c
*B. diazoefficiens* + *P. indica*	83 ± 2.19 b
*B. diazoefficiens* + *Bacillus* sp. MN54 + *P. indica*	90 ± 3.58 a

Treatment-sharing means with the same alphabetical letters were considered statistically non-significant.

### 3.2 Triple inoculation promoted plant growth and symbiotic traits of soybean under field conditions

**FIGURE 1:**
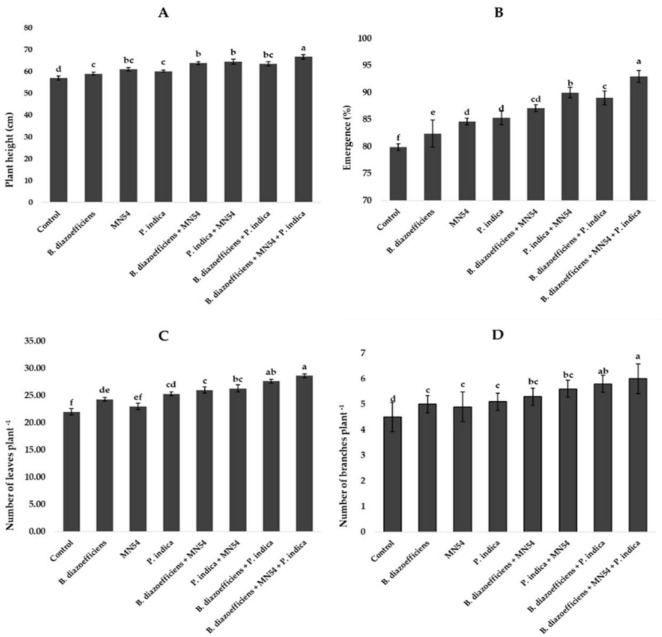
Effect of *Bradyrhizobium diazoefficiens*, *Bacillus* sp. MN54 and *Piriformospora indica* on **(A)** plant height, **(B)** emergence (%), **(C)** number of leaves plant^–1^, **(D)** number of branches plant^–1^ in soybean under field conditions. The error bars represent the least significant difference among treatments at *P* ≤ 0.05.

Inoculation significantly affected all growth parameters compared to uninoculated control (Figure 1). A maximum increase in plant height (17.01%) was observed in plants that received combined inoculations (*B. diazoefficiens*+ *Bacillus* sp. MN54 + *P. indica*) followed by *Bacillus* sp. MN54 + *P. indica* (13.15%) over uninoculated control (Figure 1A). The highest emergence (16.31%) was recorded when *B. diazoefficiens* was applied in combination with *P. indica* and *Bacillus* sp. MN54 over control (Figure 1B). Moreover, significant increases in the number of leaves and number of branches were observed in all inoculated treatments over uninoculated control, but the maximum increase in number of leaves plant^–1^ (17.83%) and number of branches plant^–1^ (20.40%) were recorded in plants that received triple inoculations over *B. diazoefficiens* application alone (Figures 1C,D).

**FIGURE 2:**
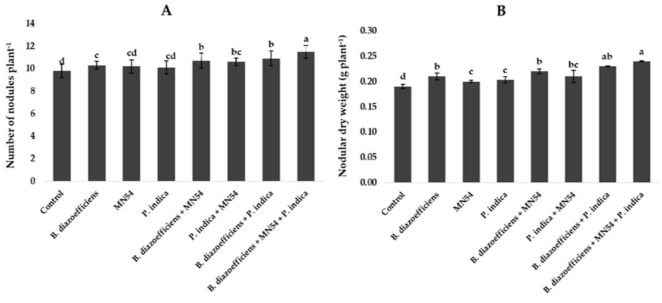
Effect of *Bradyrhizobium diazoefficiens*, *Bacillus* sp. MN54 and *Piriformospora indica* on **(A)** number of nodules plant^–1^, **(B)** nodular dry weight in soybean under field conditions. The error bars represent the least significant difference among treatments at *P* ≤ 0.05.

The results of symbiotic dependency by plants treated with individual symbionts or with triple inoculations are given in Table 2. It was noted the plants inoculated with *B. diazoefficiens* alone and in combination with *Bacillus* sp. MN54, *P. indica* inoculation showed a positive growth response over the uninoculated control. The highest symbiotic efficiency response (161.85%) was observed in plants that received triple inoculation plants (*B. diazoefficiens* + *Bacillus* sp. MN54 + *P. indica*) over control. Similarly, dual inoculation has a more pronounced effect when compared with *B. diazoefficiens* alone, but maximum response (132.95%) was observed in *B. diazoefficiens* + *Bacillus* sp. MN54 + *P. indica* treated plants over the plants solely inoculated with *B. diazoefficiens*. The highest number of nodules plant^–1^ (17.35%) was observed in triple inoculation (*B. diazoefficiens*+ *Bacillus* sp. MN54 + *P. indica*) over uninoculated control (Figure 2A). This treatment response was also higher over the application of *B. diazoefficiens* alone. Data regarding nodular dry weight was also presented in Figure 2B. It has been found *B. diazoefficiens* inoculant increased dry weight (10.52%) over control. This increase was more pronounced when *B. diazoefficiens* was combined with *P. indica* and *Bacillus* sp. MN54. A maximum increase in nodular dry weight (26.31%) was observed with triple inoculation of *B. diazoefficiens*, *P. indica*, and *Bacillus* sp. MN54 over control.

**TABLE 2:** Effect of *Bradyrhizobium diazoefficiens, Bacillus* sp. MN54 and *Piriformospora indica* inoculation on symbiotic dependency (%) of soybean under field conditions.

Treatments	Symbiotic dependency (%)	
	**Over control**	**Over *B. diazoefficiens***
*B. diazoefficiens*	121.73 ± 12.2 d	–
*B. diazoefficiens + Bacillus* sp. MN54	125.71 ± 11.4 c	103.07 ± 14.5 c
*B. diazoefficiens* + *P. indica*	150.08 ± 9.8 b	123.29 ± 17.7 b
*B. diazoefficiens* + *Bacillus* sp. MN54 + *P. indica*	161.85 ± 14.2 a	132.95 ± 25.9 a

Treatment-sharing means with the same alphabetical letters were considered statistically non-significant.

### 3.3 Triple inoculation response to yield traits of soybean under field conditions

**FIGURE 3:**
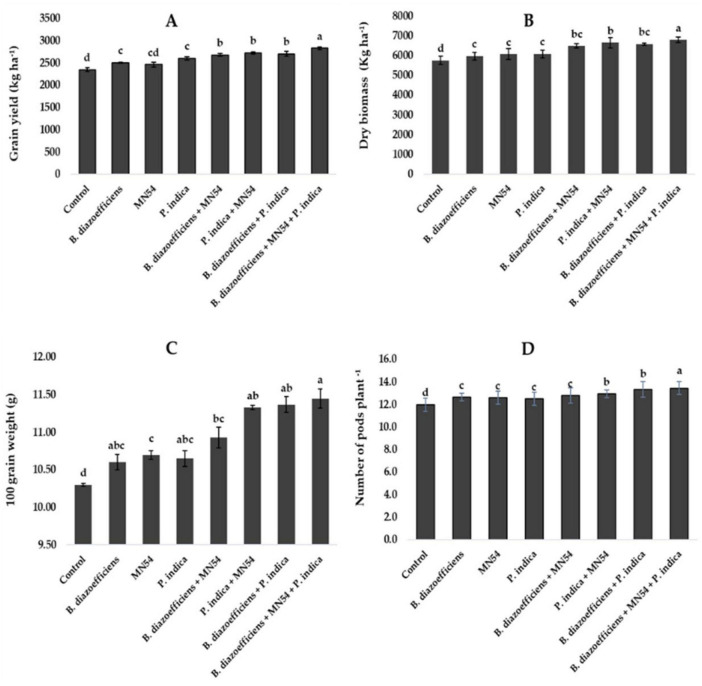
Effect of *Bradyrhizobium diazoefficiens*, *Bacillus* sp. MN54 and *Piriformospora indica* on **(A)** grain yield, **(B)** dry biomass, **(C)** 100 grain weight, **(D)** number of pods in soybean under field conditions. The error bars represent the least significant difference among treatments at *P* ≤ 0.05.

Combined inoculations significantly increased soybean grain yield over the sole application of *B. diazoefficiens* and to uninoculated control (Figure 3A). The maximum increase of 20.50% was observed in triplicate inoculation, i.e., *B. diazoefficiens* + *P. indica* and *Bacillus* sp. MN54 as compared to uninoculated control, as shown in Figure 3A. The co-inoculation of *Bacillus* sp. MN54 + *B. diazoefficiens*, and *P. indica* + *B. diazoefficiens* increased biomass (10.36 and 11.70%, respectively) over *B. diazoefficiens* alone (Figure 3B). However, maximum biomass (18.05%) was recorded in the plants that received a combined application of *B. diazoefficiens*, *Bacillus* sp. MN54 and *P. indica* as compared to uninoculated control. An increase was observed in 100 grain weight and number of pods plant^–1^. The increase of 11.16% was observed in 100-grain weight in triple inoculation over control (Figure 3C). Similarly, this treatment showed an increase in the number of pods plant^–1^ by 12.50% over the untreated control (Figure 3D).

### 3.4 Enhancement in physiological and quality traits of soybean under field conditions

**FIGURE 4:**
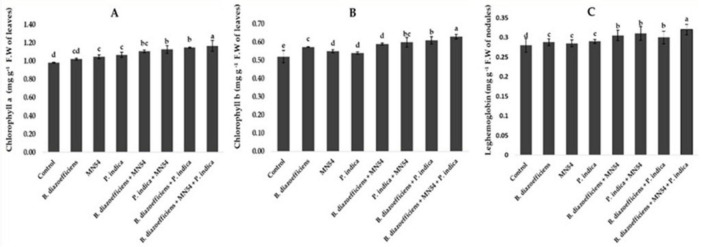
Effect of *Bradyrhizobium diazoefficiens*, *Bacillus* sp. MN54 and *Piriformospora indica* on **(A)** chlorophyll a contents, **(B)** chlorophyll b contents, **(C)** leghemoglobin contents in soybean under field conditions. The error bars represent the least significant difference among treatments at *P* ≤ 0.05.

**FIGURE 5:**
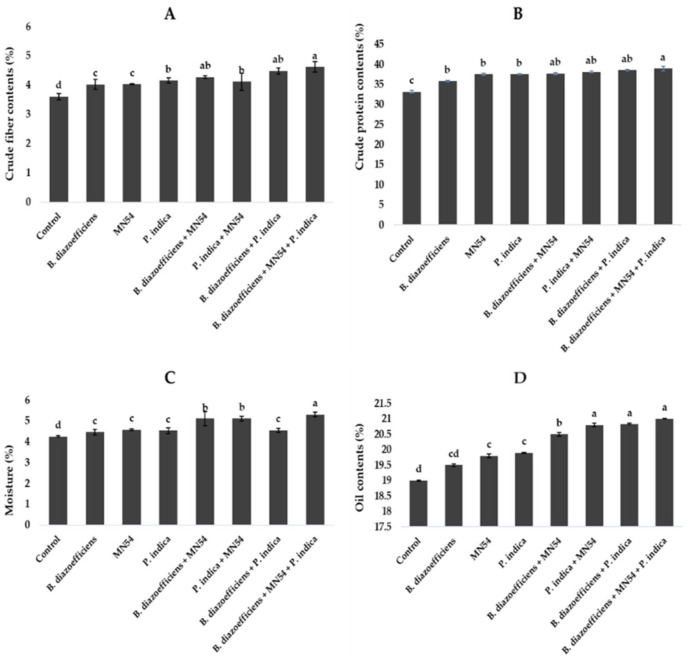
Effect of *Bradyrhizobium diazoefficiens*, *Bacillus* sp. MN54 and *Piriformospora indica* on **(A)** crude fiber contents, **(B)** crude protein contents, **(C)** moisture contents, **(D)** oil contents in soybean under field conditions. The error bars represent the least significant difference among treatments at *P* ≤ 0.05.

A significant improvement was observed in chlorophyll contents by applying single, dual, and triple inoculation over un-inoculated control (Figure 4). It has been observed that the effect of co-inoculation and triple inoculations was more pronounced over the application of *B. diazoefficiens* alone. The highest increased in chlorophyll a and b contents, i.e., 19.38 and 21.01%, respectively, were observed by application of *Bacillus* sp. MN54 and *P. indica*, along with *B*. *diazoefficiens*, compared to control (Figures 4A,B). Similarly, significant increases in leghemoglobin contents were observed in treated plants against untreated control. A 7.10 and 10.71% increase was observed by co-inoculation of *P. indica* along with *B. diazoefficiens*, and *Bacillus* sp. MN54 mixes *B. diazoefficiens*, respectively, over control. But maximum leghemoglobin contents (14.28%) were recorded in the combination of all three inoculants (*B. diazoefficiens* + *Bacillus* sp. MN54, and *P. indica*) over un-inoculated control as shown in Figure 4C. On the pooled mean basis, it has been found that a significant increase in all quality parameters has been observed in treated plants as compared to untreated (Figure 5). The co-inoculation increased quality parameters followed by single inoculation over control. The maximum increase in crude fiber, crude protein, moisture percentage, and oil contents was recorded in triplicate inoculation of *B. diazoefficiens, Bacillus* sp. MN54, and *P. indica* (14.92, 8.78, 19.28, and 10.52%, respectively) over uninoculated control (Figures 5A–D).

### 3.5 Microbial inoculations promoted the nutrient profile of soybean under field conditions

**FIGURE 6:**
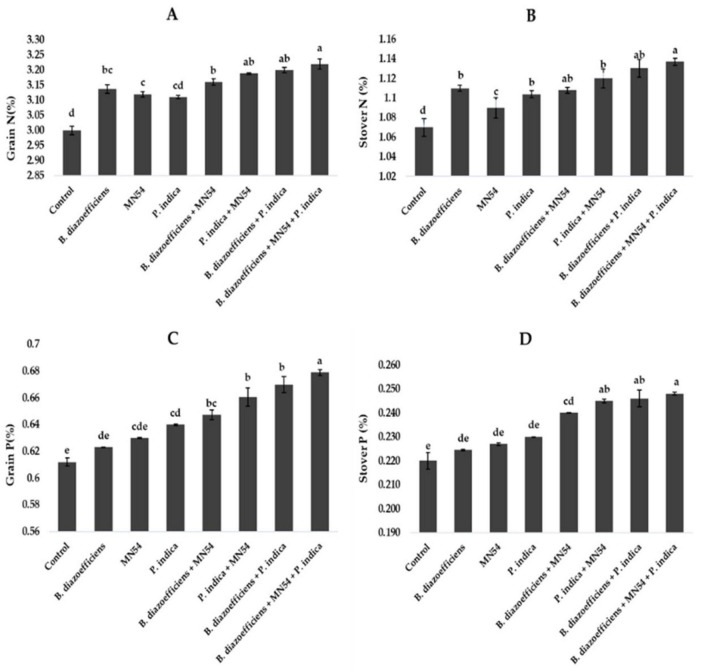
Effect of *Bradyrhizobium diazoefficiens*, *Bacillus* sp. MN54 and *Piriformospora indica* on **(A)** grain N, **(B)** stover N, **(C)** grain P, **(D)** stover P in soybean under field conditions. The error bars represent the least significant difference among treatments at *P* ≤ 0.05.

**FIGURE 7:**
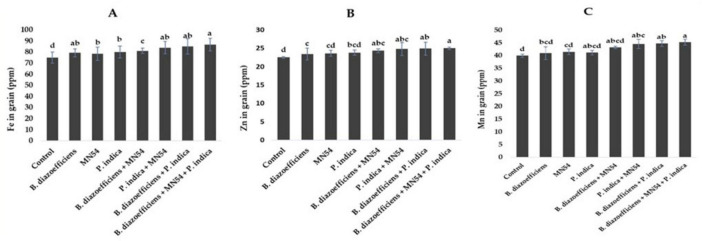
Effect of *Bradyrhizobium diazoefficiens*, *Bacillus* sp. MN54 and *Piriformospora indica* on **(A)** Fe in grain, **(B)** Zn in grain, **(C)** Mn in grain in soybean under field conditions. The error bars represent the least significant difference among treatments at *P* ≤ 0.05.

Results showed that dual and triple inoculation had a significant effect on nutrient contents in soybean over uninoculated control as shown in Figures 6, 7. Soybean plants that received triple inoculations had high grain and stover N and P contents, followed by co-inoculation as compared to untreated control and single inoculation. Maximum increases in N contents (7.33 in grain and 6.26% in stover) were observed in treatment where *B. diazoefficiens* was applied along *Bacillus* sp. MN54 and *P. indica* over uninoculated control (Figures 6A,B). Similarly, an increase in P contents was observed in the co-inoculation of *B. diazoefficiens* and *P. indica* over *B. diazoefficiens* alone. But maximum P in grain (11.31%) and stover (12.72%) was found in a combination of *B. diazoefficiens, Bacillus* sp. MN54 and *P. indica* over untreated control, as shown in Figures 6C,D. Results also showed that single, co-inoculation, and triple-inoculation had significantly improved micronutrient contents in soybean grains over uninoculated control. The highest Fe (15.60%), Zn (11.11%), and Mn (13.25%) contents were recorded in triple inoculations treatment (*B. diazoefficiens*, *Bacillus* sp. MN54, and *P. indica*) over control (Figures 7A–C).

### 3.6 Results from Pearson correlation and principal component analysis

**FIGURE 8:**
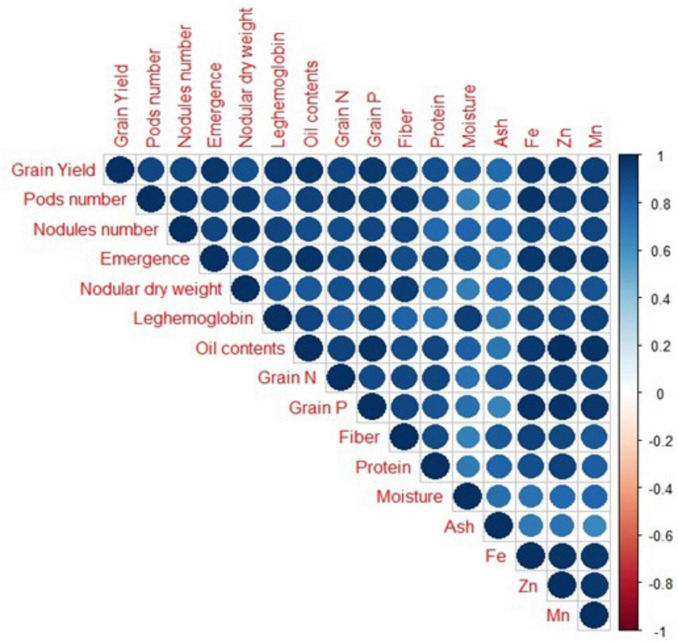
Correlation among measured parameters of soybean grown under field conditions. Positive correlations are displayed in blue color. The color intensity and the size of the circle are proportional to the correlation coefficients.

**FIGURE 9:**
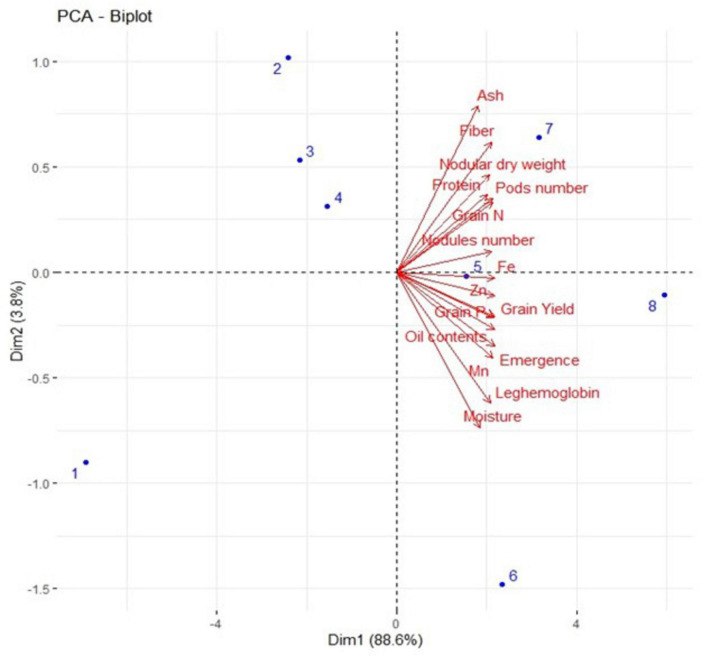
Represents the PCA biplot among measured parameters of triple inoculation in soybean under field conditions. Treatments are as T_1_. Control, T_2_. *B. diazoefficiens* inoculation, T_3_. *Bacillus* sp. MN54 inoculation, T_4_. *P. indica* inoculation, T_5_. *B. diazoefficiens* + *Bacillus* sp. MN54, T_6_. *P. indica* + *Bacillus* sp. MN54, T_7_. *B. diazoefficiens* + *P. indica*, T_8_. *B. diazoefficiens* + *Bacillus* sp. MN54 + *P. indica*.

A strong positive correlation was observed in all studied growth, yield attributes, and mineral contents of soybean crops under field conditions (Figure 8). Principal component analysis (PCA) was performed to observe interrelationships among various parameters as shown in Figure 9. The first biplot showed that among all the components, the first two components viz. PC1 (Dim1) and PC2 (Dim2) exhibited maximum contribution, accounting for 87.8% of the total dataset. Principal components 1 (Dim1) and 2 (Dim2) explained 73.9 and 13.9% of the variability among the variables studied. The distribution of all treatments showed that all inoculated treatments positively affected plant growth, yield, and nutrient contents over control. All treatments with single or co-inoculation were displaced from control. Still, the treatment where triple-inoculation was used had a more significant displacement from control, indicating a more pronounced effect.

## 4 Discussion

In this study, we found considerable improvement in the growth of soybean under the application of *B. diazoefficiens*, *Bacillus* sp. MN54, and *P. indica* either alone or in combination (Figure 1). A significant positive response was observed in plant height where treatments were applied compared to uninoculated control. This increase may be directly associated with the acceleration of plant growth by producing phytohormones (Kaur and Sharma, 2013; Aziz et al., 2020; Rafique et al., 2021). A significant increase in emergence, number of leaves per plant, and number of branches per plant were also observed with the alone application of microbes, but the combined application effect was more pronounced. According to Orrell and Bennett (2013), *Rhizobia* are critical in encouraging plants’ hostile behavior toward diseases and herbivores. This encourages and improves several growth metrics, including seed germination, emergence, seedling vigor, plant stand, root and shoot growth, and total biomass of the plants, including seed weight (Ravikumar, 2012). Studies revealed that plant growth-promoting bacteria (PGPB) exhibit a variety of traits that encourage plant growth, including the synthesis of exopolysaccharides and the solubilization of phosphate, zinc, production of indole acetic acid (IAA), and HCN. When exposed to various environmental and soil conditions, different PGPB frequently exhibit one or more features that promote plant growth (Olanrewaju et al., 2017). According to Kalam et al. (2020), numerous plant growth-promoting activities, including IAA and GA production, and P solubilization have been linked to different *Bacillus* species (Miljakovic et al., 2020; Magotra et al., 2021). Seed germination is a crucial factor and essential to generating total biomass and yield. It has been found that inoculation with *Bacillus* sp. showed increased in seedlings germination of Arachis hypogea (Shifa et al., 2015). Because they produce antibiotics, phytohormones, and the ability to solubilize phosphate, *Bacillus* sp. like *Bacillus subtilis*, *Bacillus amyloliquefaciens*, *Bacillus cereus*, *Bacillus pumilus*, and *Bacillus polymyxa* are well known for their capacity to promote plant growth and development (Majumdar, 2017). Moreover, Tsimilli-Michael and Strasser (2013) reported that *Piriformospora indica* has also been identified as a new candidate symbiont capable of delivering significant growth-promoting activity to various plants, including agricultural and medicinal crops.

In our study, all the treated plants showed increased nodule count and nodule dry weight (Figure 2). The current results are in line with the findings of Hungria et al. (2015) and Janagard and Ebadi-Segherloo (2016), who discovered that plants grown from infected seeds had more nodule growth and dry weight than plants grown from uninoculated seeds. As a result of the symbiotic interaction between *Rhizobia* and soybean plants, root nodules often start to form and expand, increasing the amount of nitrogen fixation in the plant (Tirichine et al., 2006). According to Vasileva and Ilieva (2012), rhizobia are of considerable scientific and commercial significance due to their capacity to fix atmospheric nitrogen in leguminous plants. Through synthesizing unique signal molecules known as Nod factors, rhizobia promote the development of root nodules in leguminous plants (Boundless com, 2021). *Rhizobia* produces ammonia inside the nodules, which host plants utilize as a source of absorbed nitrogen. This use significantly reduces the need for chemical fertilizers (Lin et al., 2019). According to research, the *Bacillus* sp. was able to assist the plant growth and solubilize the insoluble nutrients. According to Bhutani et al. (2018), *Bacillus* is the most prevalent non-rhizobial endophytic species in summer crops and can promote plant root development. Auxin-responsive genes are regulated by PGPR, which alters root architectural characteristics by controlling endogenous IAA levels in rice roots (Ambreetha et al., 2018). The boost in nodulation in the current study could also be attributed to higher metabolism in *P. indica*-infested plants, which have enabled them to deliver more significant amounts of carbohydrates to the *Rhizobia*. Our results are supported by earlier research (Rokhzadi and Toashih, 2011; Verma et al., 2012), which shows that PGPR can increase native rhizobia’s capacity to produce more chickpea nodules. Chickpea nodule formation may have benefited from native AMF and inoculated *P. indica* mobilizing phosphates from the soil. Our findings are consistent with those of Nautiyal et al. (2010), who showed that *P. indica* and PGPR consortia improved the ability of native rhizobia to nodulate chickpeas. According to Tavasolee et al. (2011), the opposite is true; PGPR bacterial strains decreased the fresh mass of nodules in chickpeas due to competition for photosynthetic materials.

The findings of our investigation showed that seed inoculation with *Rhizobia* boosted grain yield, biomass, and pod number; however, this reaction was further strengthened by adding *Bacillus* and *P. Indica* (Figure 3). Alam et al. (2015) revealed that soybean plants inoculated with *Rhizobia* produced more pods than uninoculated plants. It may be due to increased nitrogen availability that directly enhanced photosynthesis and biomass production. More biomass generally includes increased leaf area, which is crucial for photosynthesis and energy production, thus supporting more extensive growth and enabling the plant to support more reproductive structures, including pods. Moreover, using *Bacillus* sp. to boost *V. radiata* growth and yield proved successful. According to Kumari et al. (2018), *Bacillus megaterium*, *Bacillus pumilus*, and *Bacillus subtilis* all lengthened shoots by 55.55, 46.46, and 46.20% over control, respectively. Good plant development was seen in *Arachis hypogea* exposed to *Bacillus licheniformis* (Shifa et al., 2015). A promising method for increasing plant development and decreasing the use of toxic chemical fertilizers is to inoculate soil or crops with PGPB (Alori et al., 2012). Additionally, Meena et al. (2010) found that *P. indica*-inoculated plants had higher P contents, and Achatz et al. (2010) found that colonized plants had higher photosynthetic rates and improved production. Similarly, Ray and Valsalakumar (2010) reported that co-inoculation with *Rhizobia* and AM fungi enhanced plant vigor and nutrient uptake and significantly boosted the yield of green gram.

In our investigation, treated plants showed a substantial increase in photosynthetic pigments (Figure 4) that could be a result of the synergistic action of applied microbes, which increased the supply of N to the plant, which is a crucial structural component of chlorophyll (Zohaib et al., 2019). This study found that inoculations of *Bradyrhizobium*, *Bacillus* MN54, and *P. indica* significantly improved leghemoglobin content compared to *Bradyrhizobium* alone treatment. This might result from higher nodulation and N_2_ fixation increasing the occupancy of effective nodules, which might have increased the leghaemoglobin content. These findings fit well with the results of Kunal, and Sharma (2012), who reported a positive correlation between leghaemoglobin content and nitrogen fixation in chickpeas due to *Rhizobia* inoculation. Furthermore, one of the causes of enhanced nodulation in such plants is higher P nutrition in legumes connected to AMF. AMF are known to increase the amount of N available to legumes by promoting the growth of nodules with high levels of leghemoglobin. This might result from higher nodulation and N_2_ fixation increasing the occupancy of effective nodules, which might have increased the leghaemoglobin content.

According to the findings of our study, soybean with single, dual, and triple inoculations had higher fiber, protein, oil, and nutrient contents (Figures 5–7). The inoculation of legume crops with beneficial microbes such as *Rhizobia*, *Bacillus* spp., and *P. indica* can significantly enhance the nutrient content of the seeds, particularly protein, fat, and fiber. Each microorganism contributes uniquely through various biological mechanisms, improving plant growth, nutrient uptake, and metabolic efficiency. *Rhizobia* are best known for their role in nitrogen fixation. They form nodules on the roots of leguminous plants, where they convert atmospheric nitrogen into ammonia, making it available to the host plant. This directly enhances protein synthesis in the plant because nitrogen is a critical component of amino acids, the building blocks of proteins (Sulieman et al., 2015). Meanwhile, *Bacillus* sp. MN54 and *P. indica* have a significant role in enhancing phosphorus solubilization and nutrient uptake. These processes are crucial for efficient metabolic functioning and can increase seed oil (fat) and possibly fiber content by promoting biosynthetic pathways (Bhattacharyya and Jha, 2012; Gill et al., 2016).

In soy bean the combined effect of *R. japonicum* and *P. striata* on seed N, protein, and oil contents was significantly influenced by post-harvest plant sample analysis (Waghmare et al., 2012; Jaga and Sharma, 2015). Compared to uninoculated treatments, seed N and protein content rise due to *Rhizobia* and *Bacillus* inoculation because *Bacillus* promotes N availability and improves seed accumulation (Fatima et al., 2007). Our study’s improvement in total N content aligns with previous findings in chickpeas (Nautiyal et al., 2010) and soybean (Lima et al., 2011). This may be due to PGPB promoting root growth and root structure by increasing the number of root hairs and surface area, which thus increases plant nutrient intake and acquisition (De Sousa et al., 2021; Calvo et al., 2017; Adesemoye et al., 2008).

According to Iqbal et al. (2023), phosphate-solubilizing bacteria boost the production of auxin and cytokinin and make inaccessible P available to plants. These microorganisms improve plant P uptake and make it available in inorganic form (Qadir et al., 2024). Earlier research by Malla et al. (2004) found that *P. indica* contained significant amounts of an acid phosphatase with the potential to solubilize phosphate in the soil and deliver it to the host plant, improving P content in *P. indica* treatments. These findings are in line with our study. Moreover, the investigation has been found coherent with the results of Nautiyal et al. (2010), who reported that consortia of *P. indica* and PGPR along with native chickpea rhizobia increased the P content. PGPR might contribute to soil phosphate pool available for extraradical hyphae of AMF like fungi, to pass on to the plant, especially in soils with low P bioavailability. Furthermore, due to the ability of *P. indica* to colonize host plant roots and create a robust root system increases the soil’s ability to absorb nutrients (Hosseini et al., 2019). In addition, PGPR may contribute to the amount of soil phosphate pool available for extraradical hyphae of AMF transmitted to the plant, particularly in soils with low P bioavailability. Additionally, because *P. indica* may colonize host plant roots and develop a robust root system, the soil’s capacity to absorb nutrients increases (Hosseini et al., 2019).

## 5 Conclusion

The tripartite microbial augmentation of Bradyrhizobium diazoefficiens, *Bacillus* sp. MN54 and P. indica significantly influenced soybean growth, yield, and nutrient contents. Our findings demonstrate the substantial enhancements in crucial growth parameters, indicating the positive impact of microbial symbiosis on soybean growth. The application of the microbial consortium resulted in notable improvements in soybean yield. Importantly, our analysis of nutrient profiling revealed significant enhancements in essential nutrients such as nitrogen, phosphorus, zinc, iron, and manganese, highlighting the potential of tripartite microbial augmentation to enhance the nutritional quality of soybean crops. These findings emphasize the importance of harnessing compatible consortium inoculation for sustainable agriculture practices to improve crop productivity and nutritional value. Further research into the mechanisms underlying microbial interactions and their effects on plant-microbe-soil interactions will be valuable for optimizing microbial-based strategies in soybean cultivation and advancing agricultural sustainability.

## Data Availability

The original contributions presented in the study are included in the article/supplementary material, further inquiries can be directed to the corresponding author.

## Footnotes

1
https://jcm.brc.riken.jp/en/

2
https://www.dsmz.de/
